# Dy^3+^ and Mn^4+^ Ions Co-Doped Stannate Phosphors for Applications in Dual-Mode Optical Thermometry

**DOI:** 10.3390/molecules30071569

**Published:** 2025-03-31

**Authors:** Zaifa Yang, Zhide Wang, Yi Su, Wenyue Zhang, Yu Zheng

**Affiliations:** College of Physics and Electronic Engineering, Qilu Normal University, Jinan 250200, China; 15020303985@163.com (Z.W.); suyi051115@163.com (Y.S.); zhang111120231122@163.com (W.Z.); 18553155373@163.com (Y.Z.)

**Keywords:** optical thermometry, FIR technology, phosphor, rare earth

## Abstract

In order to break through the limitations of the application of traditional temperature measurement technology, non-contact optical temperature sensing material with good sensitivity is one of the current research hotspots. Herein, a series of Dy^3+^ and Mn^4+^ co-doping Mg_3_Ga_2_SnO_8_ fluorescent materials were prepared successfully, and the crystal structure, phase purity, and morphology of the synthesized phosphors were comprehensively investigated, as well as their photoluminescence properties, energy transfer, and high-temperature thermal stability. The two pairs of independent thermally coupled energy levels of Dy^3+^ ions and Mn^4+^ ions in Mg_3_Ga_2_SnO_8_ are utilized to realize the dual-mode optical temperature detection with excellent performance. On the one hand, based on the different ionic energy level transitions of ^4^F_9/2_→^6^H_13/2_ and ^2^E_g_→^4^A_2g_ responding differently to temperature, two emission bands of 577 nm and 668 nm were chosen to construct the fluorescence intensity ratio thermometry, and the maximum sensitivity of 1.82 %K^−1^ was achieved at 473 K. On the other hand, based on the strong temperature dependence of the lifetime of Mn^4+^ in Mg_3_Ga_2_SnO_8_:0.06Dy^3+^,0.009Mn^4+^, fluorescence lifetime thermometry was constructed and a maximum sensitivity of 2.75 %K^−1^ was achieved at 473 K. Finally, the Mg_3_Ga_2_SnO_8_: 0.06Dy^3+^,0.009Mn^4+^ sample realizes dual-mode optical temperature measurement with high sensitivity and a wide temperature detection range, indicating that the sample has promising applications in optical temperature measurement.

## 1. Introduction

In chemistry, physics, and life movement, temperature is one of the most important parameters to determine the state of matter. Accurate measurement and precise control of temperature is of great significance in people’s daily life, industrial and agricultural production, and scientific research [1,2]. Traditional contact thermometers have been widely used in many areas, but slow response time and poor ability to adapt to complex environments are the main obstacles limiting their application. In recent years, non-contact optical thermometers are getting more and more attention because they not only avoid the above problems to a large extent but also have a strong anti-interference ability and high signal resolution [3,4]. The modes of non-contact optical temperature sensors mainly include the fluorescence intensity, fluorescence intensity ratio (FIR), peak position, fluorescence lifetime (FL), and emission diffusion [5]. These modes all utilize the characteristics of the optical parameters in the luminescent material that change with temperature to establish the relationship between temperature and these parameters and then obtain the temperature of the object under test. The technique has shown great potential for use in various applications, such as temperature mapping of living cells, microelectronics, and micro-optics, owing to its fast response, high sensitivity, and high spatial resolution [6,7].

Currently, the most widely studied fluorescence temperature sensors are mainly realized by energy transfer between thermally coupled energy level pairs of rare earth ions. The energy gap between these thermally coupled energy level pairs must be less than 2000 cm^−1^ [8]. An excessively large energy level gap hinders the thermal population of electrons from lower to higher energy levels, preventing effective thermal coupling. Consequently, the temperature sensitivity of such sensors cannot exceed 2878/T^2^, resulting in relatively low sensitivity for this class of fluorescent temperature sensors [9]. In addition, the narrow energy difference between thermally coupled energy level pairs leads to the overlap of the two monitored emission peaks, which results in poor signal differentiation. The above constraints have seriously hindered the further development of thermally coupled energy level pair-type fluorescence temperature sensors [10]. Researchers have obtained an optical temperature sensor with high sensitivity by studying the emission strategies of different luminescent centers, thus effectively solving the problem of low sensitivity of fluorescent temperature sensors. For example, Zheng et al. designed a temperature sensing material system with SrB_4_O_7_:Eu^2+^/Sm^2+^, which utilized the broadband emission of Eu^2+^ and the ^5^D_0_→^7^F_0_ emission of Sm^2+^ to obtain ultrahigh temperature sensitivity [11]. Wei et al. investigated the luminescence excellence and high temperature sensitivity of Na_5_Y_9_F_32_:Ho^3+^/Yb^3+^/Eu^3+^ [12]. The double emission of Bi^3+^/Eu^3+^ was constructed in Ca_2_Sb_2_O_7_, and the difference in the thermal quenching properties of Bi^3+^/Eu^3+^ and the efficient energy transfer process between them were utilized to achieve highly sensitive temperature detection [13]. Although these materials achieve relatively good temperature sensing characteristics, the relative sensitivity needs to be further improved.

Mg_3_Ga_2_SnO_8_ (MGS) matrix materials with a cubic structure were first reported by Zhu et al. They constructed MGS substrates based on Mg_2_TiO_4_ through a co-substitution strategy of [Ga^3+^-Ga^3+^] instead of [Mg^2+^-Ti^4+^] and Sn^4+^ instead of Ti^4+^. The results show that the MGS matrix material has stable physicochemical properties and low phonon energy [14]. Therefore, the study of Dy^3+^ and Mn^4+^ co-doped MGS phosphors for optical temperature measurement has attracted our attention. In this work, the novel Dy^3+^ and Mn^4+^ co-doped MGS phosphors were synthesized in this work. This study primarily investigated the crystal structure, luminescence characteristics, and variable-temperature emission spectra of the phosphors, while analyzing the energy transfer phenomenon between Dy^3+^ and Mn^4+^. Furthermore, based on the distinct thermal quenching properties of the two dopants, we designed two different temperature measurement modes utilizing FIR and FL, achieving relative sensitivities of 1.82 %K^−1^ and 2.75 %K^−1^, respectively.

## 2. Results and Discussion

### 2.1. Crystal Structure

**Figure 1:**
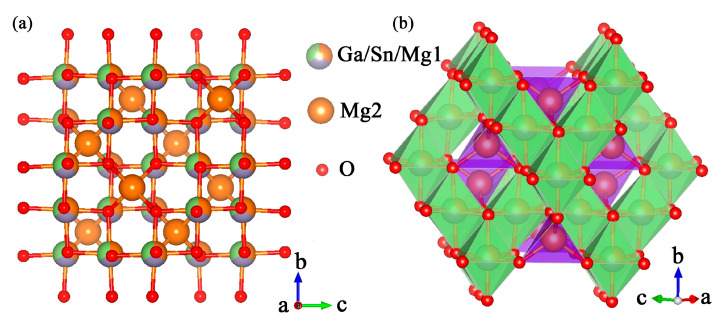
(**a**) Crystal structure of MGS. (**b**) The coordination environments of Mg, Ga, and Sn atoms.

Figure 1a shows the crystal structure of MGS, which is a cubic structure with a Fd-3m space group [15]. It can be seen that there are two different ionic occupancies for the Mg ions. Interestingly, three different Ga^3+^, Sn^4+^, and Mg^2+^ ions occupy the same sites in this cubic crystal structure. As shown in Figure 1b, the Mg(1)^2+^, Sn^4+^, Ga^3+^, and six O^2−^ are adjacent, forming an irregular octahedron, and one Mg(2)^2+^ and four O^2−^ are adjacent, forming a regular tetrahedron. The [Mg(1),Sn,GaO_6_] and [Mg(2)O_4_] are alternately connected by sharing oxygen atoms and thus form the basic skeleton of the cubic structure. In order to better study the lattice occupation of Dy^3+^ and Mn^4+^ doping into MGS, the corresponding D_r_ values of dopant ions into Ga^3+^, Sn^4+^, and Mg^2+^ positions are determined by the following equation [16]:

(1) $$D_{r}=\frac{R_{m}(CN)-R_{d}(CN)}{R_{m}(CN)}$$

where CN, R_m_(CN), and R_d_(CN) denote the coordination number, the ionic radius of the substrate, and the radius of the dopant ion, respectively. Theoretically, doping is allowed when the D_r_ value is less than 30% [17]. The ionic radii of MGS are as follows: Mg^2+^ (r = 0.57 Å, CN = 4; r = 0.72 Å, CN = 6), Ga^3+^ (r = 0.86 Å, CN = 6), Sn^4+^ (r = 0.62 Å, CN = 6), Dy^3+^ (r = 0.912 Å, CN = 6), and Mn^4+^ (r = 0.53 Å, CN = 6) [18]. It can be found that the corresponding D_r_ values of Dy^3+^ into the Ga^3+^, Sn^4+^, and Mg^2+^ positions are 5.71%, 33.95%, and 26.67%, and for Mn^4+^ are 38.37%, 14.52%, and 26.39%, respectively. Therefore, the Dy^3+^ and Mn^4+^ ions tend to occupy the Ga^3+^ and Sn^4+^ lattice positions. More precise ion substitution can be further determined by later Rietveld refinement.

**Figure 2:**
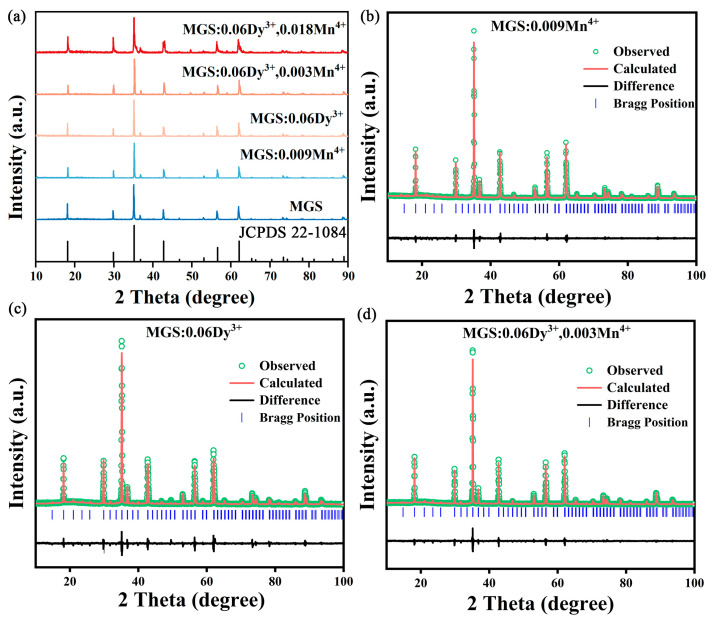
(**a**) XRD patterns of MGS matrix, MGS:0.06Dy^3+^, MGS:0.009Mn^4+^, MGS:0.06Dy^3+^,0.003Mn^4+^, and MGS:0.06Dy^3+^,0.018Mn^4+^ phosphors; XRD Rietveld refinement of (**b**) MGS:0.009Mn^4+^, (**c**) MGS:0.06Dy^3+^, and (**d**) MGS:0.06Dy^3+^,0.003Mn^4+^.

Figure 2a shows the X-ray diffraction (XRD) patterns of the MGS matrix, MGS:0.06Dy^3+^, MGS:0.009Mn^4+^, MGS:0.06Dy^3+^,0.003Mn^4+^, and MGS:0.06Dy^3+^,0.018Mn^4+^ phosphors. Comparing with the MGS standard card (JCPDS 22-1084) with the cubic phase structure, it is found that the diffraction peaks of all the samples coincide with the characteristic diffraction peaks of the standard spectrum, which indicates that the introduction of Dy^3+^ and Mn^4+^ has not changed the crystal structure of the MGS matrix, and the prepared samples are all in a pure phase [19]. The XRD data of the MGS:0.06Dy^3+^, MGS:0.009Mn^4+^, and MGS:0.06Dy^3+^,0.003Mn^4+^ samples were refined to further account for the ionic substitution of Dy^3+^ and Mn^4+^. Table 1 lists all the refined structural parameters obtained by the Rietveld refinement method. XRD refinements of MGS:0.06Dy^3+^, MGS:0.009Mn^4+^, and MGS:0.06Dy^3+^,0.003Mn^4+^ are shown in Figure 2b–d, respectively. The associated residual factors corresponding to MGS:0.009Mn^4+^, MGS:0.06Dy^3+^, and MGS:0.06Dy^3+^,0.003Mn^4+^ samples were as follows: R_wp_ = 9.6%, 10.3%, 8.8%, and R_p_ = 7.5%, 8.1%, and 6.9%, with all of the results staying at a low level. These data demonstrate that the crystal structure of the experimental samples agrees very well with the refined model.

**Figure 3:**
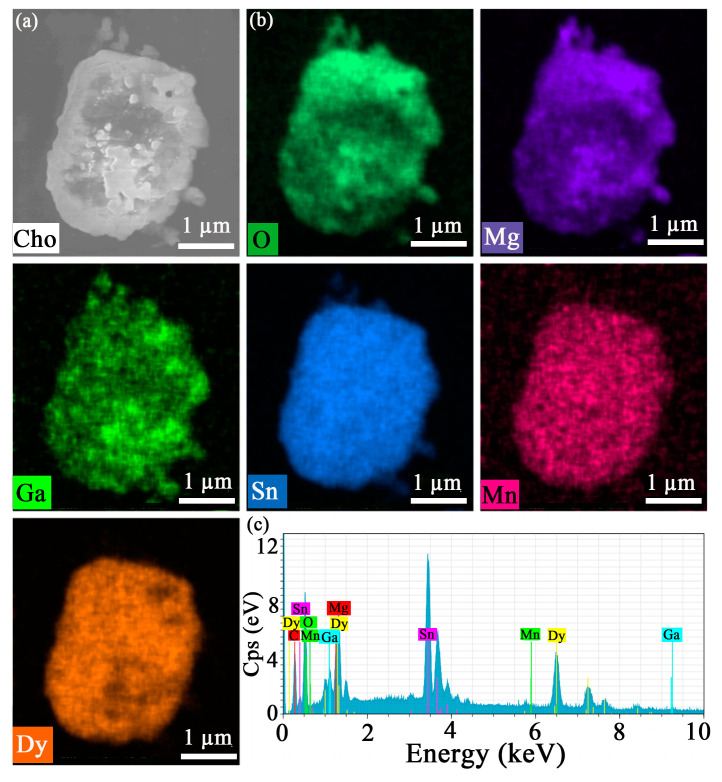
The (**a**) SEM, and (**b**) elemental mapping image for MGS:0.06Dy^3+^,0.003Mn^4+^; (**c**) the elemental distribution energy spectrum of the MGS:0.06Dy^3+^,0.003Mn^4+^.

Figure 3a shows the scanning electron microscopy (SEM) image of the MGS:0.06Dy^3+^,0.003Mn^4+^ sample, which was prepared as a microcrystal of about a few micrometers in size. And it can be seen that the prepared sample shows a slight agglomeration phenomenon, which is a relatively common result in the high-temperature solid-phase method [20]. Figure 3b shows the associated elemental mapping of the MGS:0.06Dy^3+^,0.003Mn^4+^ sample, and the results further confirm the uniform distribution of Mg, Ga, Sn, O, Dy, and Mn in the selected areas. In addition, Figure 3c shows the elemental distribution energy spectrum of the MGS:0.06Dy^3+^,0.003Mn^4+^ sample, where it can be clearly observed that the elements of Mg, Ga, Sn, O, Dy, and Mn are uniformly distributed in the randomly selected sample area, further indicating that Dy^3+^ and Mn^4+^ were successfully doped into the sample.

### 2.2. Optical Properties

**Figure 4:**
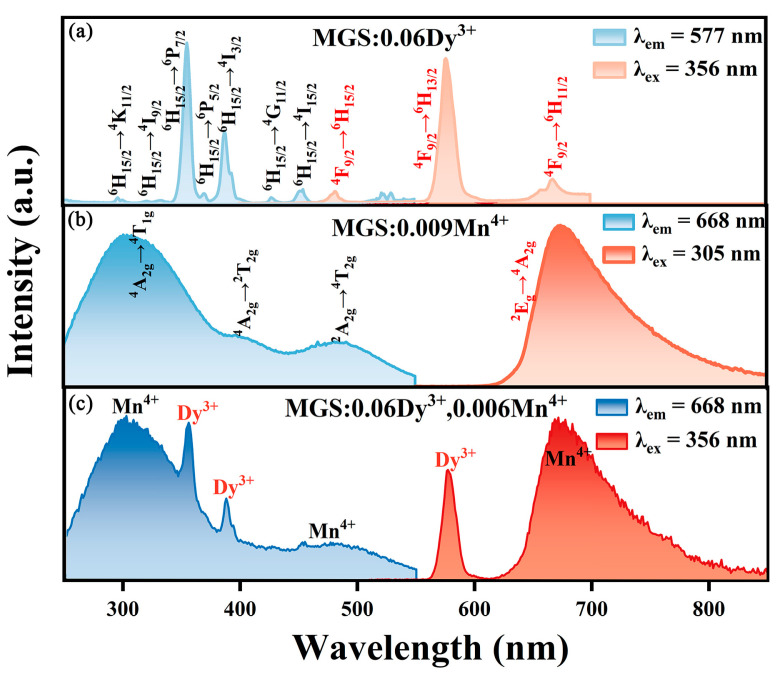
The excitation and emission spectra of (**a**) MGS:0.06Dy^3+^, (**b**) MGS:0.009Mn^4+^, and (**c**) MGS:0.06Dy^3+^, 0.006Mn^4+^.

Figure 4a shows the excitation spectrum of the MGS:0.06Dy^3+^ sample at the monitoring wavelength of 577 nm and the emission spectrum under the excitation wavelength of 356 nm. At the monitoring wavelength of 577 nm, the MGS:0.06Dy^3+^ sample shows several obvious excitation peaks at 297 nm (^6^H_15/2_→^4^K_11/2_), 322 nm (^6^H_15/2_→^4^I_9/2_), 356 nm (^6^H_15/2_→^6^P_7/2_), 371 nm (^6^H_15/2_→^6^P_5/2_), 388 nm (^6^H_15/2_→^4^I_3/2_), 428 nm (^6^H_15/2_→^4^G_11/2_), and 455 nm (^6^H_15/2_→^4^I_15/2_) [21,22]. From the emission spectrum, we can see that MGS:0.06Dy^3+^ has three main emission bands, which are located in the blue light region at 483 nm (^4^F_9/2_→^6^H_15/2_), the yellow light region at 577 nm (^4^F_9/2_→^6^H_13/2_), and the red light region at 667 nm (^4^F_9/2_→^6^H_11/2_), respectively [23]. Figure 4b displays the spectra of the MGS:0.009Mn^4+^ phosphor. The excitation spectrum of the MGS:0.009Mn^4+^ phosphor was measured to consist of three broad excitation bands located at 305, 403, and 488 nm. Among them, the two excitation peaks at 403 and 488 nm were attributed to the ^4^A_2g_→^2^T_2g_ and ^4^A_2g_→^4^T_2g_ transitions of Mn^4+^, respectively [24]. The excitation peak at 305 nm arises from the coupling of the O^2−^→Mn^4+^ charge transfer band and the ^4^A_2g_→^4^T_1g_ transition of Mn^4+^ [25]. Figure S1 also shows the excitation diagram of MGS:0.009Mn^4+^ at 77 K. Notably, a sharp R-line emission peak at about 653 nm and a small raised peak at about 670 nm can also be observed in the emission spectrum. The R-line emission peak is attributed to the zero-phonon line (ZPL) transition of Mn^4+^, which stemmed from the unperturbed octahedral sites [26]. In the figure, the emission spectrum of MGS:0.06Dy^3+^ in the range of 450–550 nm overlaps with the excitation spectrum of MGS:0.009Mn^4+^, suggesting the possibility of energy transfer between Dy^3+^ and Mn^4+^. Figure 4c shows the spectra of the MGS:0.06Dy^3+^,0.006Mn^4+^ phosphor. The emission peaks of the MGS:0.06Dy^3+^,0.006Mn^4+^ sample consisted of the characteristic emission peaks of Dy^3+^ and Mn^4+^. In addition, the excitation peaks were mainly from the characteristic peak of Mn^4+^, while two characteristic peaks of Dy^3+^ were also shown at 356 nm and 388 nm. These results indicate that there may be energy transfer from Dy^3+^ to Mn^4+^.

**Figure 5:**
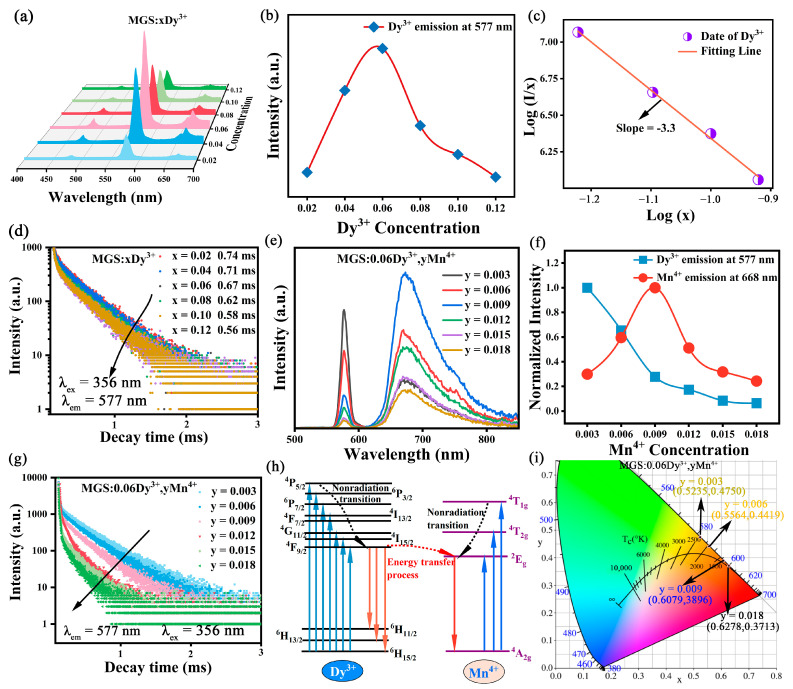
(**a**) The emission spectra of MGS:xDy^3+^ (0.02 ≤ x ≤ 0.12); (**b**) integrated emission intensity at 577 nm with Dy^3+^ dopants; (**c**) fitting line of lg(I/x) versus lg(x) in MGS:xDy^3+^ (0.06 ≤ x ≤ 0.12); (**d**) decay curves of MGS:xDy^3+^ (0.02 ≤ x ≤ 0.12) phosphors excited by 356 nm; (**e**) the emission spectra of MGS:0.06Dy^3+^,yMn^4+^ (0.003 ≤ y ≤ 0.018); (**f**) integrated emission intensity at 577 nm and 668 nm with Mn^4+^ dopants; (**g**) decay curves of MGS:0.06Dy^3+^,yMn^4+^ (0.003 ≤ y ≤ 0.018) excited by 356 nm; (**h**) schematic diagram of energy transfer process from Dy^3+^ to Mn^4+^; (**i**) CIE chromaticity diagrams of MGS:0.06Dy^3+^,yMn^4+^ (0.003 ≤ y ≤ 0.018).

As shown in Figure 5a, the shape of the emission spectra of MGS:xDy^3+^ (0.02 ≤ x ≤ 0.12) phosphors under 356 nm excitation is almost unaffected, but the luminescence intensity of the samples exhibits significant differences. Figure 5b shows the relationship between the relative luminous intensity of MGS:xDy^3+^ (0.02 ≤ x ≤ 0.12) and the doping concentration of Dy^3+^. The concentration quenching of MGS:xDy^3+^ (0.02 ≤ x ≤ 0.12) phosphors starts to appear at the doping concentration x = 0.06. Before that, the luminous intensity of the sample increases with the increase in the doping concentration of Dy^3+^. And when the concentration quenching occurs, the luminescence intensity decreases with the further increase in the doping concentration of Dy^3+^. In general, the quenching phenomenon of phosphor concentration is mainly caused by the energy transfer between active ions [27]. And the energy transfer mechanism can be determined by calculating the critical distance (R_c_) between activated ions. If the R_c_ is greater than 5 Å, then electric multipolar interactions dominate; if the R_c_ is less than 5 Å, then exchange interactions dominate. According to Blasse’s theory, the R_c_ was determined by the following equation [28]:

(2) $$R_{c}=2{\left(\frac{3V}{4\pi x_{c}N}\right)}^{1/3}$$

where V is the cell volume of MGS, x_c_ is the critical concentration (x_c_ = 0.06), and N refers to the number of cations in MGS. According to the XRD refinement results, V = 604.85 Å^3^, and N = 8. The critical distance R_c_ for MGS:xDy^3+^ (0.02 ≤ x ≤ 0.12) phosphors is 20.11 Å according to the calculation of Equation (2). Exchange interactions require that the distances between activated ions are less than 5 Å, so it can be hypothesized that the electro-multipolar interactions between the Dy^3+^ are dominant. In addition, the multipolar interactions can be further categorized into dipole–dipole (d-d), dipole–quadrupole (d-q), and quadrupole–quadrupole (q-q) interactions [29]. For the concentration quenching caused by the above three kinds of electric multipole interactions, the relationship can be inferred using the following equation [30]:

(3) $$\frac{I}{x}=K{\left(1+\beta {\left(x\right)}^{Q/3}\right)}^{-1}$$

where I is the luminescence intensity of the sample, x is the doping concentration of the activating ions, K and Q are constants, and Q is taken as 6, 8, and 10 for d-d, d-q, and q-q interactions, respectively. Figure 5c shows the curve of lg(I/x) versus log(x) for the MGS:xDy^3+^ samples, and it can be seen that lg(I/x) is almost linear with log(x), and its slope is -3.3 after fitting. The resulting value of Q is calculated to be 9.9, which is closer to 10. Therefore, the concentration quenching in the MGS:xDy^3+^ (0.02 ≤ x ≤ 0.12) phosphors can be attributed to the q-q interaction. The decay curves of MGS:xDy^3+^ (0.02 ≤ x ≤ 0.12) phosphors were measured under 356 nm excitation with the ^4^F_9/2_→^6^H_13/2_ transition emission of Dy^3+^ (577 nm) as the monitoring wavelength, as shown in Figure 5d. The decay curves of all the samples slightly deviated from the mono-exponential and decayed with a biexponential function, which conformed to the following equation [31]:

(4) $$I=I_{0}+A_{1}\exp(-t/\tau_{1})+A_{2}\exp(-t/\tau_{2})$$

where t is the time, I_(t)_ is the luminous intensity of the sample at the time t, A_1_ and A_2_ are constants, and τ_1_ and τ_2_ are the lifetime in two decay forms, respectively. When the decay curve is a double exponential function of the decay, the average lifetime can be expressed as follows:

(5) $$\tau =(A_{1}\tau_{1}^{2}+A_{2}\tau_{2}^{2})/(A_{1}\tau_{1}+A_{2}\tau_{2})$$

The average lifetimes of the samples at each doping concentration were calculated according to Equation (5), which showed that the lifetime of the samples decreased slightly with the increase in the Dy^3+^ doping concentration.

To explore the effect of Mn^4+^ content on luminescence properties, the emission spectra of MGS:0.06Dy^3+^,yMn^4+^ (0.003 ≤ y ≤ 0.018) samples are shown in Figure 5e. Apparently, the peak shapes and peak positions of the Dy^3+^ and Mn^4+^ emission peaks did not change significantly; only the intensities changed significantly. The variation in the intensity of the emission peak of Dy^3+^ and Mn^4+^ with respect to that of the Mn^4+^ doping concentrations is given in Figure 5f. The intensity of Dy^3+^ (577 nm) decreases monotonically with the increase in the Mn^4+^ doping concentration, while the intensity of the emission peak of the Mn^4+^ ion at 668 nm first increases, confirming the existence of the energy transfer from Dy^3+^ to Mn^4+^. To further confirm the existence of the energy transfer phenomenon, Figure 5g shows the decay curves of MGS:0.06Dy^3+^,yMn^4+^ (0.003 ≤ y ≤ 0.018) samples. The decay curves at different Mn^4+^ doping concentrations can all be fitted with a double exponential, and the corresponding fitting calculations are presented in Table 2. It can be seen that the lifetime monotonically decreases with the increase in the Mn^4+^ concentration. This result also provides strong evidence for the existence of energy transfer. In order to present the process of energy transfer more clearly, Figure 5h plots the energy levels of Dy^3+^ and Mn^4+^ in the MGS:0.06Dy^3+^,yMn^4+^ (0.003 ≤ y ≤ 0.018) samples. Apparently, the mechanism and pathway of energy transfer between the two ions are clearly presented in the figure.

Color coordinates are indicators used to qualitatively measure the color of light, generally using the CIE color coordinate system implemented by the International Commission on Illumination in 1931 [32]. By analyzing the luminescence spectra of MGS:0.06Dy^3+^,yMn^4+^ (0.003 ≤ y ≤ 0.018) phosphors at an excitation wavelength of 356 nm, their CIE coordinates were determined and presented in Table 2. The CIE chromatogram of the MGS:0.06Dy^3+^,yMn^4+^ (0.003 ≤ y ≤ 0.018) samples are labeled in Figure 5i. The results show that there is a significant change in the CIE coordinates for Mn^4+^ concentration in the 0.003 to 0.018 range. Specifically, the coordinates move from (0.5235, 0.4750) to (0.6106, 0.3863). And the emission color of MGS:0.06Dy^3+^,yMn^4+^ (0.003 ≤ y ≤ 0.018) phosphors gradually changed from yellow to orange and finally to red. Moreover, the CIE coordinates of all samples are located at the edge of the coordinate plot indicating that all samples have very high color purity.

**Figure 6:**
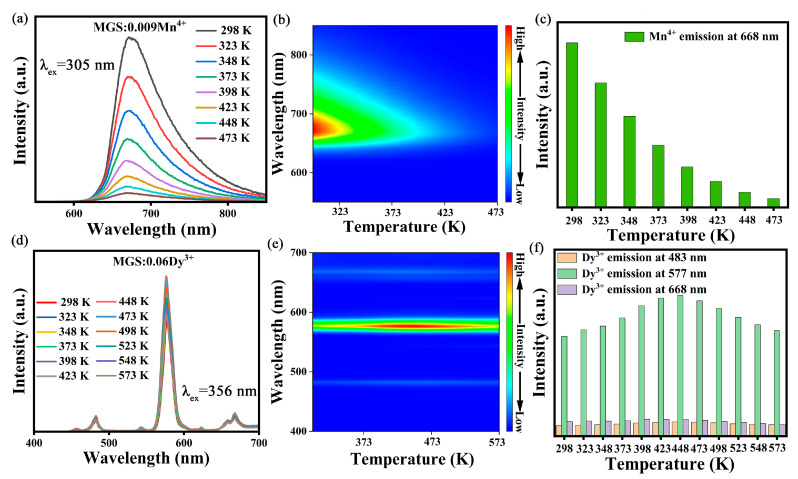
(**a**) The temperature-dependent emission spectra and (**b**) the corresponding contour maps of MGS:0.009Mn^4+^; (**c**) integrated intensities of Mn^4+^ with temperature; (**d**) the temperature-dependent emission spectra, and (**e**) the corresponding contour maps of MGS:0.06Dy^3+^; (**f**) integrated intensities of Dy^3+^ with temperature.

The temperature-dependent emission spectra of the MGS:0.009Mn^4+^ phosphor under 305 nm excitation between 298 and 473 K are shown in Figure 6a. And Figure 6b shows the color-filled contour plot of the normalized variable temperature emission spectrum of the MGS:0.009Mn^4+^ sample. Figure S2 demonstrates the normalized distribution of the temperature-dependent emission spectra of MGS:0.009Mn^4+^. The ^2^_Eg_→^4^A_2g_ transition emission of Mn^4+^ does not show a significant peak shift with increasing temperature due to the small electro-phonon coupling between the ^2^E energy level and the ^4^A_2_ energy level [33]. The full width at half maximum of MGS:0.009Mn^4+^ broadens from 76 nm to 84 nm as the temperature increases from 298 K to 473 K. This is due to the weakening of the field strength of the expanded lattice at higher temperatures in the Mn^4+^ ionic crystal field. Figure 6c demonstrates the variation in the integral intensity of the variable temperature emission spectrum of the MGS:0.009Mn^4+^ sample. As the temperature increases, the luminescence of MGS:0.009Mn^4+^ undergoes a significant decay due to the thermal quenching phenomenon [34]. The MGS:0.009Mn^4+^ samples maintained 55.4% of the room temperature luminous intensity at 348 K and 15.6% of the room temperature luminous intensity at 423 K, and when the temperature is greater than 473 K, the luminescence of MGS:0.009Mn^4+^ is close to complete quenching. The emission spectra and relative emission contour spectra of MGS:0.06Dy^3+^ with temperature changes are shown in Figure 6d,e. The peak shape and peak position of the Dy^3+^ emission peak did not change significantly with increasing temperature. Figure 6f illustrates the variation in the emission peaks of Dy^3+^ normalized by the initial value of the integral intensity with the process of temperature increase. The luminescence intensity increases and then decreases as the test temperature is increased in steps of 25 K. In general, the release of electrons from defect-induced trap states at elevated temperatures is strongly correlated with an increase in the intensity of the sample. The integrated intensity of MGS:0.06Dy^3+^ at 573 K can still maintain about 99.1% of the emission intensity at 298 K. Therefore, it can be used as a two-probe fluorescence intensity ratio temperature sensing parameter based on the relative changes in luminescence intensity of Dy^3+^ and Mn^4+^ ions.

### 2.3. Temperature Sensing Characterization

**Figure 7:**
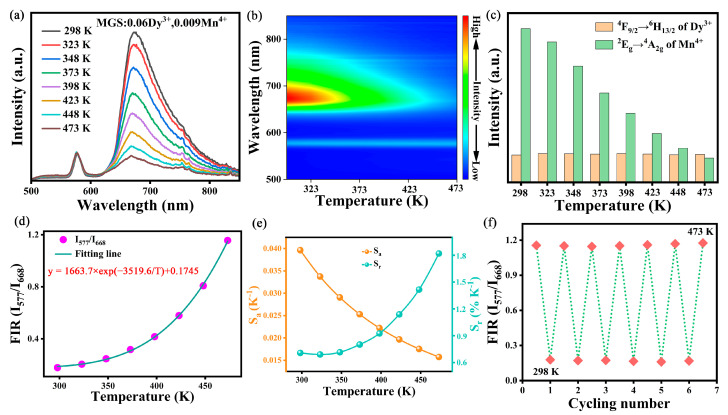
(**a**) The temperature-dependent emission spectra and (**b**) the corresponding contour maps of the MGS:0.06Dy^3+^,0.009Mn^4+^ phosphor; (**c**) integrated intensities of Dy^3+^ and Mn^4+^ for the MGS:0.06Dy^3+^,0.009Mn^4+^ phosphor with temperature; (**d**) temperature-dependent FIR values from I_577_/I_668_ of MGS:0.06Dy^3+^,0.009Mn^4+^; (**e**) calculated S_r_ and S_a_ at different temperatures by FIR; (**f**) FIR temperature-cycling values of I_577_/I_668_ with 6 cycles of heating and cooling processes.

In order to further investigate the performance of this phosphor for optical temperature sensing application and to explore its potential application in temperature sensing, Figure 7a,b shows the emission spectra and corresponding two-dimensional images of the MGS:0.06Dy^3+^,0.009Mn^4+^ phosphor under excitation at 356 nm for the tested temperatures from 298 K to 473 K. The emission intensity of the emission peak of Dy^3+^ located near 577 nm (^4^F_9/2_→^6^H_13/2_) is basically unchanged when the temperature is gradually increased, while the emission intensity of Mn^4+^ located at 668 nm (^2^E_g_→^4^A_2g_) is rapidly bursting with the increase in temperature, as shown in Figure 7c. Therefore, the FIR model can be constructed for temperature measurement based on the fact that the luminescence intensity of the two ions responds differently to temperature at different temperatures. The relationship between FIR (I_577 nm_/I_668 nm_) and temperature can be described by the following equation [35]:

(6) $$FIR=\frac{I_{Dy}}{I_{Mn}}=\frac{1+A_{Dy}\exp(-\Delta E/(kT))}{1+A_{Mn}\exp(-\Delta E/(kT))}\approx A\exp(-B/T)+C$$

where T is the absolute temperature; A, B, and C are the relevant constant parameters. In the field of luminescence temperature measurement, absolute sensitivity (S_a_) and relative sensitivity (S_r_) are parameters necessary for the performance of the measured temperature, which are calculated as follows [36]:

(7) $$S_{a}=\left|\frac{\partial FIR}{\partial T}\right|=A\exp(-\Delta E/(kT))\times \frac{\Delta E}{kT^{2}}$$

(8) $$S_{r}=100\%\left|\frac{\partial FIR}{FIR\partial T}\right|=\frac{100\%A\exp(\frac{-\Delta E}{kT})}{C+A\exp(\frac{-\Delta E}{kT})}\times \frac{\Delta E}{kT^{2}}$$

The fitting line of the FIR value to the temperature change is shown in Figure 7d, and the fitting accuracy R^2^ reaches 99.94%, which indicates that the fitting results are very reliable and lays a foundation for further investigation of the temperature sensing performance of the phosphor. The relationship between the FIR (I_577 nm_/I_668 nm_) value and temperature can be expressed by the fitting result as: FIR (I_577 nm_/I_668 nm_) = 1663.7×exp(-3519.6/T) + 0.1745. And the fitted curves of S_a_ and S_r_ versus temperature calculated by Equations (7) and (8) are shown in Figure 7e, respectively. From the figure, the value of S_r_ increases gradually with increasing temperature and reaches 1.82 %K^−1^ at 473 K. The value of S_a_ decreases from 0.04 K^−1^ to 0.016 K^−1^ with increasing temperature and has a maximum value of 0.04 K^−1^ at 298 K. Compared to the optical thermometry phosphors already reported in Table 3, the MGS:0.06Dy^3+^,0.009Mn^4+^ phosphor has a wider thermometry range and higher sensitivity. For temperature sensing materials, cycling stability is also an important parameter to measure its performance; the change in FIR value with temperature during multiple temperature cycling is shown in Figure 7f, and the FIR value can be restored to the initial state after six temperature cycles, indicating that the phosphor has good reversibility and reliability in temperature sensing. In conclusion, the MGS:0.06Dy^3+^,0.009Mn^4+^ phosphor has good temperature sensing performance, and can be regarded as an optical material with potential value for optical temperature sensing.

**Figure 8:**
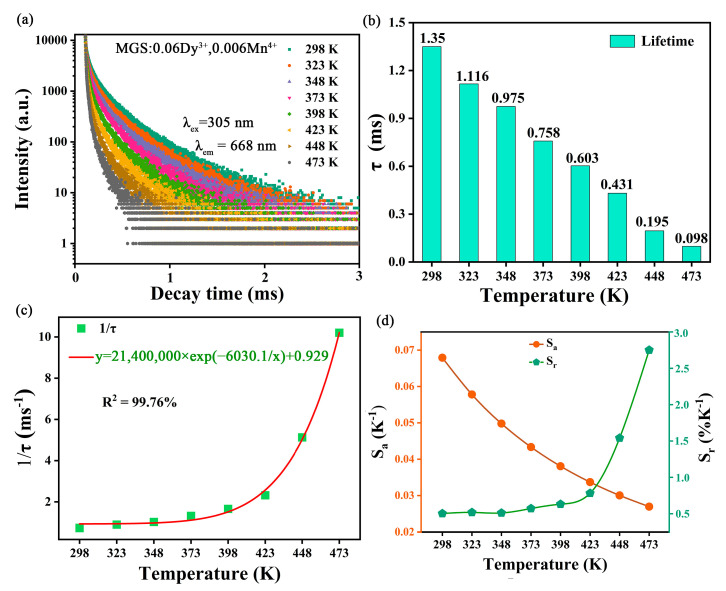
(**a**) The decay curves of MGS:0.06Dy^3+^,0.009Mn^4+^ at different temperatures excited by 305 nm; (**b**) the lifetime of Mn^4+^ at different temperatures; (**c**) fitting curve of temperature-dependent FL; (**d**) the S_a_ and S_r_ based on FL of Mn^4+^.

The use of fluorescence lifetime for temperature sensing is another very promising measurement option, which has the inherent advantage of calibration-free measurements, independent of external factors such as sample size and fluctuations in excitation power. Figure 8a shows the decay curves of Mn^4+^ in the MGS:0.06Dy^3+^,0.009Mn^4+^ sample as a function of temperature. As shown in the figure, the lifetime of Mn^4+^ ions decreases significantly with increasing temperature. As shown in Figure 8b, the lifetime of the MGS:0.06Dy^3+^,0.009Mn^4+^ sample decreases rapidly from 1.35 ms to 0.098 ms when the temperature is increased from 298 to 473 K. And the lifetime was fitted to the temperature for temperature sensing characterization, and was calculated according to the following Mott-Seitz equation [37]:

(9) $$\frac{1}{\tau_{\left(T\right)}}=\frac{1}{\tau_{0}}(1+D\exp(-\Delta E\prime /(kT))$$

where τ_(T)_ is the lifetime corresponding to a given temperature T, and τ_0_ is the lifetime at 298 K. The fitted curve of lifetime with temperature obtained by the above equation is shown in Figure 8c, which has a high degree of fit of 99.76%, thus it can be determined that the MGS:0.06Dy^3+^,0.009Mn^4+^ sample has a high degree of accuracy in temperature sensing. Its temperature-sensing sensitivity was further examined, and Sa and Sr were defined with the following equation [38]:

(10) $$S_{a}=\left|\frac{\partial \tau }{\partial T}\right|$$

(11) $$S_{r}=100\%\left|\frac{1}{\tau }\frac{\partial \tau }{\partial T}\right|$$

The S_a_ and S_r_ values for FL thermometry were calculated from the above equation and plotted as shown in Figure 8d. Apparently, a maximum value of 2.75 %K^−1^ was obtained for S_r_ at 473 K and 0.068 K^−1^ for S_a_ at 298 K. The maximum relative sensitivities S_r_ for FL thermometry in different materials reported in the recent literature are demonstrated in Table 3. Compared to these materials, the MGS:0.06Dy^3+^,0.009Mn^4+^ sample exhibits superior temperature sensitivity. Therefore, the MGS:0.06Dy^3+^,0.009Mn^4+^ phosphor has potential applications in the field of FL thermometry.

**Table 3:** S_r_-Max of some rare earth ions and transition metal ions with co-doped phosphors based on FIR or FL mode.

Sample	Temperature Range (K)	S_r_-Max (% K^−1^)	Mode	Ref.
La_2_LiSbO_6_:Mn^4+^,Dy^3+^	303–523	0.769	FIR	[10]
Ca_2_LaNbO_6_:Mn^4+^,Eu^3+^	298–498	1.51	FIR	[39]
SrLaLiTeO_6_:Mn^4+^,Dy^3+^	298–673	1.6	FIR	[40]
MGS:0.06Dy^3+^,0.009Mn^4+^	298–473	1.82	FIR	This work
Ca_2_GdSbO_6_:Eu^3+^, Mn^4+^	303–503	1.47	FL	[22]
Ba_2_GdNbO_6_:Eu^3+^, Mn^4+^	303–483	1.73	FL	[41]
MGS:0.06Dy^3+^,0.009Mn^4+^	298–473	2.75	FL	This work

## 3. Materials and Methods

### 3.1. Preparation of Materials

The MGS:xDy^3+^ (0.02 ≤ x ≤ 0.12), MGS:0.06Dy^3+^,yMn^4+^ (0.003 ≤ y ≤ 0.018), and MGS:0.009Mn^4+^ phosphors were synthesized by a high-temperature solid-phase reaction. And the raw materials of (MgCO_3_)_4_·Mg(OH)_2_·5H_2_O (99.5%), Ga_2_O_3_ (99.99%), SnO_2_ (99.5%), Dy_2_O_3_ (99.99%), and MnCO_3_ (99.5%) were weighed according to the same molar ratios as the chemical formulae and uniformly milled in the onyx mortar for 30 min. Then, the mixture was transferred into a corundum crucible and pre-sintered at 650 °C for 4 h in a box furnace. After the pre-sintered sample cooled to room temperature, it was removed and ground again for 5–10 min. The ground sample was then placed in a corundum crucible and reacted in air at 1450 °C for 6 h in the box furnace. After cooling to room temperature, the sample was removed and ground into a powdered sample for subsequent testing.

### 3.2. Characterization of Materials

The crystalline phase structure of the samples was determined by using a Cu-targeted Kα-radiation powder X-ray diffractometer (XRD, D8 Advanced, Bruker, Karlsruhe, Germany) with a scanning step of 0.02°. The surface morphology of the samples was analyzed by field emission scanning electron microscopy (SEM, JEOL JSM-IT500A, Tokyo, Japan). The excitation light source was a xenon lamp, and a variable-temperature accessory (Tian Jin Orient—KOJI Instrument, Tianjin, China) was used for variable-temperature testing. For lifetime measurement, a microsecond lamp was selected as the light source.

## 4. Conclusions

In this paper, a series of MGS:Dy^3+^,Mn^4+^ phosphors for optical temperature measurement were prepared by the high-temperature solid-phase reaction method. The microstructure, luminescence performance, and temperature sensing performance of the phosphors were studied in detail. The microstructures were analyzed by X-ray diffraction tests and refinement, indicating that the prepared phosphors feature a cubic structure with the Fd-3m space group. The luminescence performance of MGS:xDy^3+^ materials with different doping concentrations under 356 nm excitation was investigated, and the optimal doping concentration of Dy^3+^ in the phosphor was determined to be 0.06. The emission spectrum of MGS:0.06Dy^3+^,yMn^4+^ (0.003 ≤ y ≤ 0.018) indicates the presence of energy transfer of Dy^3+^→Mn^4+^ ions in the phosphors. The temperature-dependent luminescent properties of this phosphor were investigated, laying the foundation for the application of optical temperature sensors. The performance of MGS:0.06Dy^3+^,0.009Mn^4+^ as an optical temperature sensor was evaluated by FIR, with a relative sensitivity maximum of 1.82 %K^−1^ (473 K) when using the FIR (I_577 nm_/I_668 nm_) to characterize temperature. In addition, applying FL optical thermometry, the MGS:0.06Dy^3+^,0.009Mn^4+^ phosphor has a maximum value of S_r_ of 2.75 %K^−1^ (473 K) in the temperature range of 298~473 K. These results indicate that MGS:Dy^3+^,Mn^4+^ phosphors have good research and application prospects in the field of temperature sensing.

## Figures and Tables

**Table 1:** The detailed refinement results of MGS, MGS:0.009Mn^4+^, MGS:0.06Dy^3+^, and MGS:0.06Dy^3+^,0.003Mn^4+^ samples.

Sample	MGS	MGS:0.009Mn^4+^	MGS:0.06Dy^3+^	MGS:0.06Dy^3+^,0.003Mn^4+^
Space group	Fd-3m	Fd-3m	Fd-3m	Fd-3m
Symmetry	cubic	cubic	cubic	cubic
a/b/c, Å	8.4570	8.4552	8.4573	8.4566
V, Å^3^	604.85	604.46	604.92	604.77
Z	8	8	8	8
α = β = γ °	90	90	90	90
R_wp_	8.5	9.6	10.3	8.8
R_p_	6.7	7.5	8.1	6.9
χ^2^	2.25	2.83	3.20	2.32

**Table 2:** The τ, color purity, and CIE coordinates of MGS:0.06Dy^3+^,yMn^4+^ (0 ≤ y ≤ 0.018) phosphors.

Samples	τ (ms)	CIE	Color Purity (%)
MGS:0.06Dy^3+^	0.67	(0.4925, 0.4770)	88.3
MGS:0.06Dy^3+^,0.003Mn^4+^	0.52	(0.5235, 0.4750)	97.2
MGS:0.06Dy^3+^,0.006Mn^4+^	0.44	(0.5564, 0.4419)	97.8
MGS:0.06Dy^3+^,0.009Mn^4+^	0.41	(0.6278, 0.3713)	99.1
MGS:0.06Dy^3+^,0.012Mn^4+^	0.29	(0.6079, 0.3896)	98.9
MGS:0.06Dy^3+^,0.015Mn^4+^	0.21	(0.6134, 0.3836)	98.5
MGS:0.06Dy^3+^,0.018Mn^4+^	0.14	(0.6106, 0.3863)	98.7

## Data Availability

The research data are available from the authors on request.

## Supplementary Materials

The following supporting information can be downloaded at: www.mdpi.com/article/10.3390/molecules30071569/s1, Figure S1. The excitation spectrum of MGS:0.009Mn^4+^ at 77 K; Figure S2. Temperature-dependent normalized emission spectra of MGS:0.009Mn^4+^.
