# Supplementary material for: Dy3+ and Mn4+ Ions Co-Doped Stannate Phosphors for Applications in Dual-Mode Optical Thermometry
Source: Molecules. 2025 Mar 31;30(7):1569. doi: 10.3390/molecules30071569 (PMC11990255; doi:10.3390/molecules30071569)
Supplement: Supplementary file 1 [file molecules-30-01569-s001.zip › molecules-3523882-supplementary.pdf]

Supporting information

Article

Dy^{3+} and Mn^{4+} Ions Co-Doped Stannate Phosphors for Applications in Dual-Mode Optical Thermometry

Zaifa Yang *, Zhide Wang, Yi Su, Wenyue Zhang and Yu Zheng

College of Physics and Electronic Engineering, Qilu Normal University, Jinan 250200, China; 15020303985@163.com (Z.W.); suyi051115@163.com (Y.S.); zhang111120231122@163.com (W.Z.); 18553155373@163.com (Y.Z.)

* Correspondence: yangzaifa@qlnu.edu.cn; Tel.: +86-1066778147

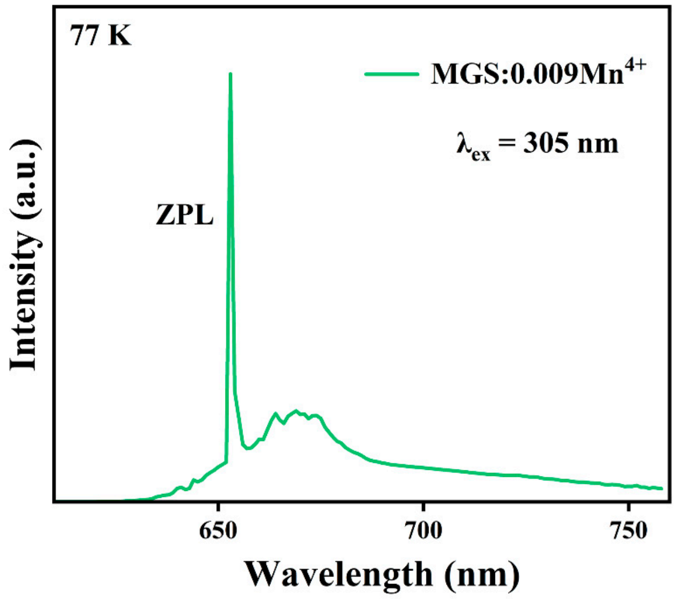

Figure S1 The excitation spectrum of MGS:0.009Mn^{4+} at 77 K.

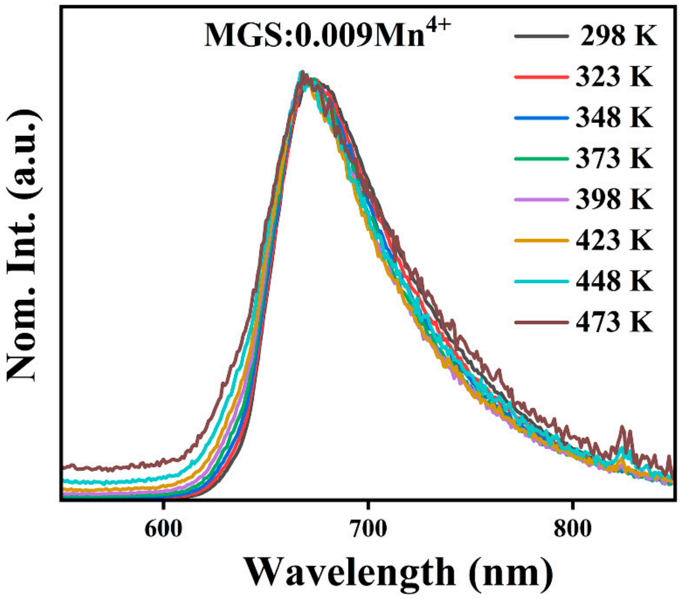

Figure S2 Temperature-dependent normalized emission spectra of MGS:0.009Mn^{4+}.
